# Classification of Genes and Putative Biomarker Identification Using Distribution Metrics on Expression Profiles

**DOI:** 10.1371/journal.pone.0009056

**Published:** 2010-02-04

**Authors:** Hung-Chung Huang, Daniel Jupiter, Vincent VanBuren

**Affiliations:** Department of Systems Biology and Translational Medicine, Texas A&M Health Science Center College of Medicine, Temple, Texas, United States of America; University of California San Diego, United States of America

## Abstract

**Background:**

Identification of genes with switch-like properties will facilitate discovery of regulatory mechanisms that underlie these properties, and will provide knowledge for the appropriate application of Boolean networks in gene regulatory models. As switch-like behavior is likely associated with tissue-specific expression, these gene products are expected to be plausible candidates as tissue-specific biomarkers.

**Methodology/Principal Findings:**

In a systematic classification of genes and search for biomarkers, gene expression profiles (GEPs) of more than 16,000 genes from 2,145 mouse array samples were analyzed. Four distribution metrics (mean, standard deviation, kurtosis and skewness) were used to classify GEPs into four categories: predominantly-off, predominantly-on, graded (rheostatic), and switch-like genes. The arrays under study were also grouped and examined by tissue type. For example, arrays were categorized as ‘brain group’ and ‘non-brain group’; the Kolmogorov-Smirnov distance and Pearson correlation coefficient were then used to compare GEPs between brain and non-brain for each gene. We were thus able to identify tissue-specific biomarker candidate genes.

**Conclusions/Significance:**

The methodology employed here may be used to facilitate disease-specific biomarker discovery.

## Introduction

It is becoming increasingly clear that the bistability (or, more generally, multistability) phenomenon found in switch-like genes is an important recurring theme in development and cell signaling [1]. Numerous synthetic gene circuits have been created in the past decade, including bistable switches, oscillators, and logic gates [2]. Bistability may be of particular relevance to biological systems that transition between discrete states (e.g., embryo maturation via positive feedback loop), that generate oscillatory responses (e.g., mitosis via negative feedback loop), or that remember transitory stimuli (e.g., cell differentiation via hysteresis) [1], [3]–[5]. Thus, it is crucial to be able to identify switch-like genes and other categories of gene expression to assist in the construction of gene regulatory networks. Additionally, distinguishing between genes with on- or off- transcriptional states and genes with rheostatic expression offers an important contribution to computational modeling efforts, including the appropriate application of Boolean network theory for gene regulatory network simulation [6]–[9].

**Figure 1:**
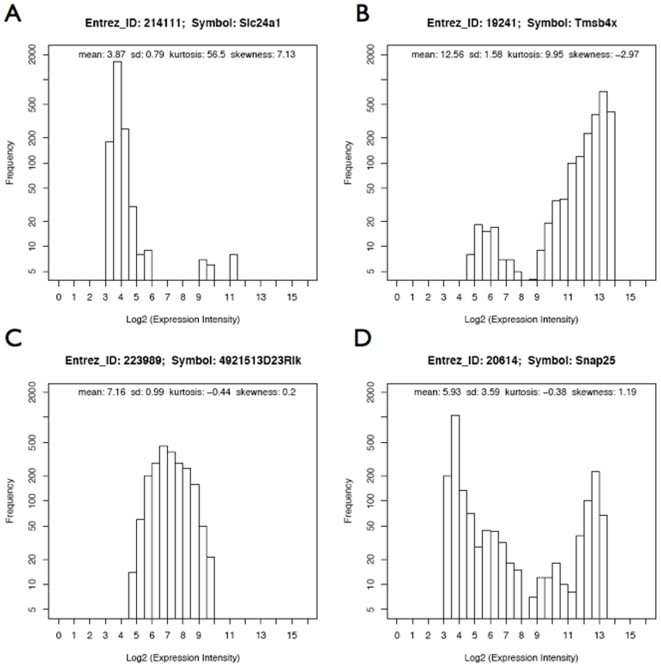
Frequency histogram plot for the expression intensity profile of genes in four categories. (a) *predominantly-off,* (b) *predominantly-on*, (c) *graded* (rheostatic), and (d) *multistable* (switch-like). The Y-axis is in log scale.

Expression profiles of more than 16,000 genes from 2,145 mouse microarray experiments were analyzed. We define the *gene expression profile* (GEP) of a gene as the distribution of the log_2_ values of normalized signal intensity across the set of studied arrays. According to visual inspection of the GEP histograms, we proposed that there were four major classes of gene expression profiles. These classes are *predominantly off*, *predominantly on*, *graded* (rheostatic), or *multistable* (the largest portion of which are bistable) switch-like gene expression profiles (**Figure 1**). In an effort to automatically assign genes to these four classes, genes were clustered according to four metrics describing the distribution characteristics of expression profiles over the large heterogeneous collection of microarray experiments described above. This work provides a foundation for the systematic classification of gene expression profiles via mining the vast resource of publicly available microarray data.

Although blood serum tests are one of the least invasive diagnostic procedures, tissue biopsy tests are commonly seen in the medical diagnosis field. Some biopsies, however, have been replaced by less invasive procedures, e.g., primary care physicians frequently perform lumbar puncture, as cerebrospinal fluid (CSF) combined with blood analyses are invaluable diagnosis windows to the diseases in the central nervous system (CNS) [10]–[13]. It has also been suggested that PCR of CSF samples should be able to replace brain biopsies for some infection tests [14]. Other examples of the analysis of biomarkers in bodily fluids include prenatal genetic tests via amniocentesis that extracts amniotic fluid from around the fetus (as an indirect test of fetus tissue) [15], [16], and liver function tests via blood sample, which test for the presence of liver enzymes, e.g., ALT(GPT), AST(GOT), ALP, GGT, and LDH [17], [18]. Thus, serum biomarkers for disease states have become increasingly important to the diagnosis and treatment of disease [19]–[23].

In addition to the classification of gene expression profiles, this report identifies a list of tissue-specific biomarker candidate genes. These candidates are expected to be useful for directly assaying the tissue of interest for transcript or expressed protein abundance. Additionally, this list provides a narrowed field of candidates of gene products or metabolites that may be measurable in patient's blood serum for diagnosis and prognosis purposes. To identify candidate biomarkers, we leveraged our studies of gene expression profiles to find genes with differential behavior between, e.g., brain and non-brain tissue samples. By identifying genes that are specifically expressed in brain, a preliminary list of candidate biomarkers for neurological disorders can be generated. We further analyzed this list to look for known biomarkers as a means of validating our approach, and performed literature reviews to identify promising candidates with potential for secretion into the blood and passage though the blood-brain barrier.

## Methods

### Data Preprocessing

A dataset that was compiled from publicly available data and used in a previous study (by Jupiter and VanBuren) [24] of more than 16,000 genes represented on 2,145 mouse array experiments conducted on Affymetrix GeneChip® mouse genome 430 2.0 arrays was analyzed in this work to study gene expression profiles. Briefly, raw microarray data was collected from NCBI's Gene Expression Omnibus (GEO) [25], [26], and features on the array were mapped to NCBI Entrez Gene IDs using Version 9 of the mapping provided by Dai *et al.* [27], yielding 16,297 gene probe sets and 64 Affymetrix control gene probe sets. The arrays were normalized using the justRMALite package [28] in BioConductor [29], which performs quantile normalization, positive match only adjustment, and Tukey median polish. A complete listing of the GSE data series on the Affymetrix platform used (GEO accession GPL1261) in the present study is given in **Table S1**.

### Metrics Used to Evaluate the GEP: Mean, SD, Kurtosis, and Skewness

Kurtosis as used here is defined as **μ**_4_ **/σ** ^4^ - 3, where **μ**_4_ is the fourth moment about the mean and **σ** is the standard deviation. Subtracting 3 gives the so-called “excess kurtosis”, which sets the kurtosis of a Normal distribution equal to zero. Kurtosis is a measure of the “peakedness” of a distribution (e.g., “gene expression intensity”), relative to a Normal distribution. A distribution with a tall peak has high kurtosis value. Skewness is a measure of the asymmetry of a distribution. A left-skewed distribution has a long left tail, and negative skewness (e. g., the GEP profile for the predominantly-on gene, **Figure 1b**). A right-skewed distribution has a long right tail, and positive skewness (e. g., the GEP profile for the predominantly-off gene, **Figure 1a**).

As kurtosis and skewness effectively describe the shape of distributions, they are good parameter choices for clustering distributions into profiles according to shape properties. Kurtosis and skewness of a GEP are expected to be useful metrics for the classification of predominantly-on and predominantly-off genes, given the high peaks and high skewness of these proposed classes of genes. Those metrics, however, do not give a good description the central position of the distribution along the *x* axis, nor do they fully describe the spread of the data. The standard deviations (s.d.) of the expression intensities for switch-like genes (e.g., those with a distinct bi-modal distribution) and rheostatic genes (e.g., those with a mono-modal and relatively unskewed distribution) will differ even though the means may be similar. Hence, s.d. is a good metric to separate rheostatic from switch-like genes. Mean expression intensity is also expected to facilitate grouping genes into one of three groups: *predominantly-off*, *rheostatic & switch-like*, and *predominantly-on*, based on their mean intensities at low, middle, and high ranges respectively. Therefore, these four metrics (mean, s.d., kurtosis, and skewness) describing GEPs were used as the metrics and parameters to cluster all the genes in the studied mouse arrays.

**Figure 2:**
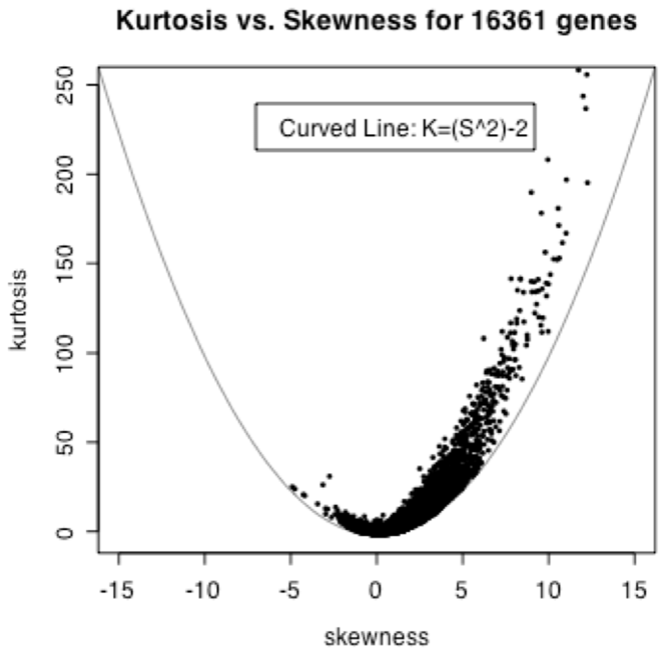
Kurtosis (K) vs.Skewness (S) plot for the GEPs of 16,361 features in 2,145 mouse arrays. Curved line is for the boundary curve with the equation of K = S^2^−2.

The joint distribution of unscaled kurtosis and skewness among the GEPs under study is non-random (**Figure 2**). This is apparent from the asymmetry of the skewness distribution, as a random distribution of skewness would have roughly as many genes with negative skewness as genes with positive skewness. Although the positive skew seen in this dataset is likely due to the underlying biological heterogeneity of the data, it should be noted that some normalization procedures may affect skew in this way [30]. Positive skew from normalization using RMA is not expected here, but we have not shown this explicitly.

### Log_2_ Transformation of Kurtosis and Normalized Scaling of Four Metrics

Before performing cluster analysis, we normalized and rescaled the distributions of our four metrics, across all the GEPs. This is done to ensure that the data is on a scale that is amenable to clustering, and so that each metric has a similar weight and contribution to the clustering. For three of the metrics, the normalization and rescaling was trivial; mean, standard deviation and skewness were re-scaled to the range −50 to 50. Excess kurtosis, however, ranges between -2 and infinity, and for this reason it is useful to log_2_-transform this parameter, in order to “spread out” the low end of this distribution. This was done after adding 2 to the excess kurtosis, insuring that we are taking the logarithm of positive numbers. The log_2_-transformed excess kurtosis was then scaled to the range −50 to 50 as for the other three metrics (Note: this range is arbitrary). The results of normalization and scaling can be seen in **Figure S1**.

### Clustering of the Genes into Four Categories

Honoring our proposition that there are four major classes of gene expression profiles, we clustered the genes by K-Means clustering (KMC) with k = 4 based on the mean, standard deviation, kurtosis, and skewness of the expression intensity profile for each gene. The resulting four clusters were expected to contain genes that corresponded to characteristics belonging to the following four categories: *graded* (rheostatic), *switch-like*, *predominantly-on* or *predominantly-off*.

### Silhouette Validation Method

To validate cluster membership, the silhouette validation technique [31] was used to calculate the silhouette width for each gene in our analyses. A complete description of this method is provided in **File S1**. The genes with silhouette value less than 0 were removed from each cluster; the remaining genes in each cluster were then designated to belong to one of the four categories as described above.

### Gene Function Enrichment Analysis

Each class of genes defined by our final clustering was further analyzed using the WebGestalt Gene Set Analysis ToolKit [32]. This allowed us to identify KEGG pathways and Gene Ontology (GO) terms that are enriched within each class of genes.

### Tissue-specific Studies and Biomarker Identification

The arrays used in this study assay a heterogeneous selection of tissues and biological states. This heterogeneity may confound results. For example, a switch-like gene may be predominantly-on in some tissues, and *predominantly-off* in some other tissues; another *switch-like* gene may have both high and low expression states in some tissues, while it has exclusively high or low expression states in other tissues. Additionally, *predominantly-on* or *predominantly-off* genes are likely to be interesting with respect to the exceptional cases in specific tissues (i.e. the cases where *predominantly-on* genes are ‘off’, and the cases where *predominantly-off* genes are ‘on’). With these ideas in mind, we further examined GEPs after grouping the arrays by the tissue type of the hybridized sample. In addition, strong candidates for tissue-specific biomarker genes are expected to have distinctive GEP in the tissue of interest when compared to the GEP derived from other tissues.

**Figure 3:**
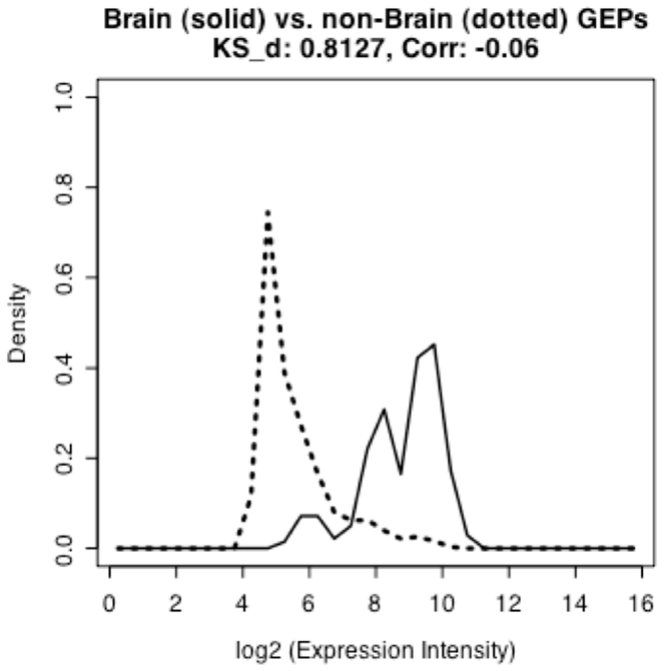
A selected representative example comparing the GEPs of *Grla3* in brain (solid line) and non-brain (dashed line) tissues.

Source information for the samples hybridized to the 2,145 arrays under study was obtained from the GEO web site [33]. Samples from brain, lung, liver, embryo, heart, and small intestine were among the most abundant sample types in the dataset. A preliminary visual assessment of the tissue specificity of a GEP for a given gene was provided by a simple examination of GEP plots, overlaid side by side, from the tissue of interest and all other tissues. Tissue specificity for each gene was systematically assessed using the Kolmogorov-Smirnov distance (KS_d) [34], [35] and Pearson correlation coefficient (Corr) [36], [37] computed between the two compared GEPs (i.e., these measures are used to compare a gene's GEP in a specific tissue of interest to that gene's GEP obtained from all other tissues). The most promising tissue-specific biomarker candidate genes should have a large KS_d and low magnitude Corr values between the two GEPs (**Figure 3**). The most promising tissue-specific biomarker candidate genes were thus selected with combined cutoffs for the KS_d and Corr values.

## Results and Discussion

### Clustering of Mouse Genes into Four Profile Categories

**Figure 4:**
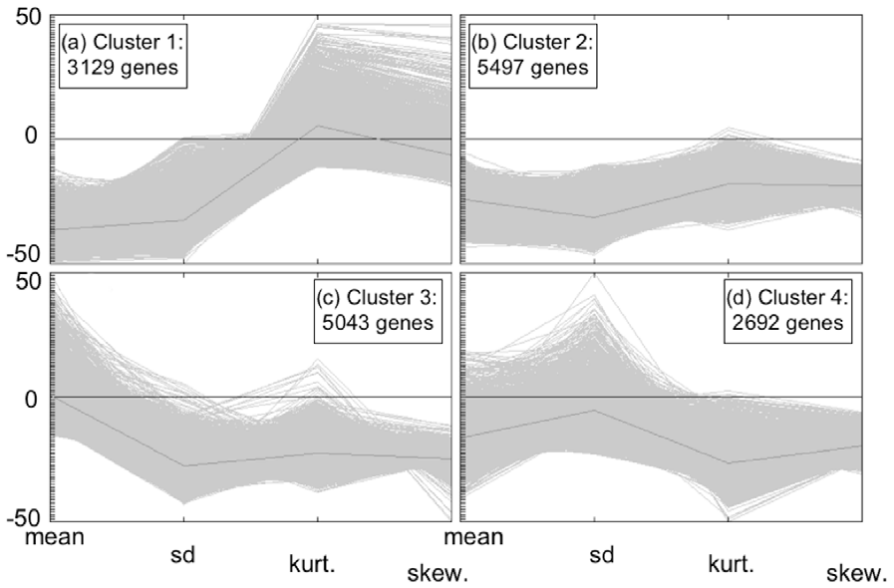
GEP metrics for each gene clustered into one of four clusters. Gene categories: (a) predominantly-off, (b) graded, (c) predominantly-on, and (d) switch-like genes.

K-Means Clustering (KMC) with k = 4 was used to make preliminary groupings (**Figure 4**) based on the four GEP metrics described in Methods. Genes in cluster 1 have low mean and high kurtosis values reflecting the weak expression intensities and high peak in GEP for predominantly-off genes. Genes in cluster 2 have regular (i.e., not extreme) metric values reflecting characteristics for graded genes. Genes in cluster 3 have high mean values reflecting the strong expression intensities for predominantly-on genes. On average, predominantly-on genes have lower kurtosis than the predominantly-off genes. This is reflective of the broader tails in predominantly-on GEPs, which in turn suggests a relaxed exclusivity of the regulatory program for predominantly-on genes as compared with predominantly-off genes. The genes in cluster 4 have regular mean and high s.d. values reflecting the characteristics for switch-like genes. Thus, the resulting four clusters represent approximately the four categories of gene expression profiles proposed above (**Figure 1**). **Figure 4** shows that most of the genes can be categorized approximately into one of the four kinds described in **Figure 1**. However, given the small number of classes we propose, it is expected that there will be ambiguously classified genes in the clusters, so we used each gene's silhouette value next to evaluate the confidence that a gene belongs to a particular class.

### Refinement of Gene Categories by Silhouette Values of GEP Metrics

The silhouette values for the genes in each cluster were calculated (see Methods). The distributions of the silhouette values in each cluster can be viewed in **Figure S2**. The genes with negative silhouette value were removed from each cluster. **Table 1** lists the number of genes with *Si*>0 in each cluster (category) that were used in further analysis. **Table S2** lists all CEL files used in the analysis by GEO accession, the resulting raw and scaled distribution metrics, and annotated lists of refined cluster assignments.

**Table 1:** The number of genes in each cluster (category).

	Cluster 1	Cluster 2	Cluster 3	Cluster 4
Initial	3129	5497	5043	2692
Si >0	2951	5484	5008	2388
Known Genes*	2944	5478	5004	2387

* Numbers of “Known Genes” that were reported by the WebGestalt gene set analysis toolkit.

### Pathway Enrichment for 2,944 Predominantly-Off Genes

In cluster 1 (*the predominantly-off* gene class), there are 2,944 known genes reported by WebGestalt for the KEGG pathway enrichment analysis. As seen in **Table 2**, “Complement and coagulation cascade” had the lowest reported *p*-value among the significantly enriched pathways [38]. Theses pathways are active in circulating blood. As the array data under study here did not include serum or plasma samples, it is reasonable that the enriched genes in the “Complement and coagulation cascade” were categorized as *predominantly-off*. Although the classification of these genes as *predominantly-off* is expected to be a correct classification for these genes, it must be noted that samples where these genes are expected to be transcribed were simply not present in the dataset. This example underscores the importance of considering the proposed classifications as provisional, and that each classification is sensitive to the limited diversity represented in the samples studied.

**Table 2:** Significantly enriched KEGG pathways for *predominantly-off* genes (*p*< = 0.01).

KEGG pathway	O: Genes observed	1E: Genes expected	2R: Ratio of enrichment	3p-Values< = 0.01
Complement and coagulation cascade	28	10.85	2.58	3.48e-7
Arachidonic acid metabolism	25	11.39	2.19	4.74e-5
Metabolism of xenobiotics by cyrochrome P450	21	9.04	2.32	7.10e-5
Linoleic acid metabolism	17	6.87	2.47	1.35e-4
Neuroactive ligand-receptor interaction	68	44.67	1.52	1.49e-4
C21-Steroid hormone metabolism	7	1.81	3.87	4.51e-4
Maturity onset diabetes of the young	12	4.52	2.65	5.98e-4
Cell communication	30	17.00	1.76	8.34e-4
Cytokine-cytokine receptor interaction	55	36.71	1.50	9.20e-4
Stilbene, Coumarine and lignin biosynthesis	5	1.09	4.61	9.84e-4

1 The 3rd column gives the expected number of genes in a given KEGG pathway (This is equal to the total number of genes in the selected set multiplied by the total number of genes in the reference set that belong to the KEGG pathway divided by the total number of genes in the reference set).

2 The 4th column is the ratio of enrichment for the KEGG pathway (R = O/E).

3 In the 5th column, P is the *p*-value given by the Hypergeometric test.

The enriched “Linoleic acid metabolism” and “Arachidonic acid metabolism” pathways are involved in hormone protein biosynthesis and inflammatory processes [39]–[43]. Linoleic acid is an essential fatty acid which must be supplied in food. One of the products in “Linoleic acid metabolism” is arachidonic acid which is a precursor in the production of eicosanoids like Prostaglandins and Leukotrienes (in “Arachidonic acid metabolism”) [44]–[47]. Arachidonic acid is esterified into the phospholipid fats in the cell membrane. In response to many inflammatory stimuli, phospholipase is generated and cleaves this fat, releasing arachidonic acid as a free fatty acid to be further modified to form eicosanoids. Eicosanoids like Prostaglandins and Leukotrienes are potent mediators of inflammation. Prostaglandins are produced by most soft tissues. Therefore, “Linoleic acid and Arachidonic acid metabolisms” should not be confined to a limited number of tissues. Classification of these genes as *predominantly-off* thus implies that some genes for these pathways are expected be in the off state under normal conditions. For example, as seen in our result (**Figure S3**, “Linoleic acid metabolism”), the cleavage of Lecithin (i.e., phosphatidylcholine) in the cell membrane by phosphalipase A2 (EC: 3.1.1.4) [48] to release linoleic acid (Linoleate) is expected to be blocked most of the time as the gene (PLA2g1b) encoding a subunit for phosphalipase A2 is *predominantly-off* in our result. If the gene for this phospholipase was not transcriptionally off most of the time, the gene product (phosphalipase A2) would be expected to promote decomposition of the cell membranes in multiple soft tissues, which under normal conditions would have a deleterious effect on cellular health.

### Pathway Enrichment for 5,478 Graded Genes

In cluster 2, the *graded* (rheostatic) gene class, 5,478 known genes were reported by WebGestalt for KEGG pathway enrichment analysis. There were only four significantly enriched KEGG pathways from this set of genes as shown in **Table 3**. Genes for the “Neuroactive ligand-receptor interaction” pathway group were enriched with the lowest *p*-value for this class of genes. This is notable, as the *predominantly-off* gene class was also enriched for this collection of pathways, as seen in previous section. Some genes (68 genes; **Table 2**) in the “Neuroactive ligand-receptor interaction” collection of pathways are thus regulated to be in *predominantly-off* state while a larger number of genes (125 genes; **Table 3**) in this pathway group are controlled in a more *graded* manner. In our results, most of the significantly enriched and graded genes for “Neuroactive ligand-receptor interaction” encode for the miscellaneous receptor proteins located on the membrane, which belong to the transmembrane G-protein coupled receptors (GPCRs) superfamily. GPCRs are known to allow a graded signal response to fluctuating extracellular stimuli [49]. This points to a relationship between the graded transcriptional activity (encoding for the above-mentioned GPCRs) and the graded signal response (for those neuroactive ligand-receptor interactions).

**Table 3:** The only four significantly enriched KEGG pathways for graded genes (*p*< = 0.01).

KEGG pathway	O: Genes observed	^1^E: Genes expected	^2^R: Ratio of enrichment	^3^p-Values< = 0.01
Neuroactive ligand-receptor interaction	125	83.12	1.50	2.35e-8
Axon guidance	58	39.37	1.47	2.59e-4
Hedgehog signaling pathway	28	17.16	1.63	1.43e-3
Taste transduction	17	9.42	1.80	3.01e-3

Column legend is the same as in **Table 2**.

Axon guidance (second pathway in **Table 3**) is a neural development process by which neurons send out axons to reach the correct targets [50]. Growing axons have a highly motile growing tip (growth cone) sniffing out the extracellular signals (guidance cues) for which way to grow. These signals can attract or repel axons. In our results, the enriched graded genes in the “Axon guidance” pathway have been found to include genes encoding for the following three important classes of axon guidance molecules and their receptors.

1. Neutrins and the receptors, DCC and UNC5 [51], [52]: Neutrins are secreted molecules that can attract or repel axons.
2. Ephrins and the Eph receptors (Ephs) [53], [54]: Ephrins are cell surface molecules that activate Eph receptors on the surface of other cells with an attractive or repulsive interaction.
3. Semaphorins and the Plexin receptors [55]: Semaphorins are primarily axonal repellents.

The gene for another axon guidance molecule, Slit [56], was also found to be *graded*-like in our results, although the genes for its receptors (Robo class receptors) were not. As our results show that most of the components between the axon guidance molecules and their receptors are encoded by *graded* genes, we can infer a graded response for axon growth via the axon guidance pathway. Such a graded response does exist and it is best understood by the interaction of Ephrin ligands and their receptors (Ephs) which can be described by a topographic mapping model with gradients for guidance in a field of neurons such as the retina [57], [58]. In this model, a gradient of Eph receptor was expressed in retina with the anterior cells expressing very low levels and the posterior cells expressing the highest levels of the receptor. Likewise, in the optic tectum (i.e., the target of the retinal cells) of the brain, Ephrin ligands are organized in a similar gradient: high posterior to low anterior. In this manner, axons from different areas of retina can appropriately project to specific areas in the tectum in brain. This is a major feature of nervous system organization, particular in sensory systems.

The “Hedgehog signaling pathway” (third pathway in **Table 3**) provides cells with developmental programming via differential concentrations of the hedgehog signaling proteins – the Hedgehog homologues (HH) [59]–[61]. Sonic hedgehog homolog (SHH) is the best studied one of three proteins in the mammalian hedgehog family; the other two being desert hedgehog (DHH) and Indian hedgehog (IHH). SHH plays a key role in regulating vertebrate organogenesis and remains important in the adult by controlling cell division of adult stem cells and has been implicated in development of some cancers [62]–[66]. SHH is also a prominent example of a morphogen molecule that diffuses to form a concentration gradient and has different effects on the cells of the developing embryo in a concentration-dependent manner. In our results, most of the genes for “Hedgehog signaling pathway” are graded genes including the ones encoding for SHH and IHH homologues, and the transmembrane protein called Patched (PTCH) [67] which is the target for HH to bind on the cell surface.

The pathway for “Taste transduction” (fourth pathway in **Table 3**; **Figure S4**) shows a typical example that the sensory stimuli from the environment impinge on receptors, which respond by producing receptor potentials, and in turn lead to the generation of action potentials which carry information substantial distances to the brain [68]–[72]. In a taste bud, the taste is thus converted into an electrical signal sent to the brain. A receptor potential is often produced by sensory transduction with a depolarizing event resulting from inward current flow. Like the photo perception in the visual pathway [73], the ion-dependent release of neurotransmitter is graded with respect to the presynaptic membrane potential (i.e., receptor potential). A receptor potential is a form of graded potential [73]–[76]. This is reflected in our results, where genes encoding most of the ion channels and taste receptors on the membrane of taste receptor cells were found to be *graded* (**Figure S4**).

We found the common factor in these four enriched pathways described above is a graded signal response of ligand-receptor interaction in a gradient environment. These genes are regulated to control graded gene expression for ligands or receptors related to the graded response of the cell to environmental signals or stimuli.

### Pathway Enrichment for 5,004 Predominantly-On Genes

In cluster 3, the *predominantly-on* gene class, there are 5,004 known genes that were analyzed using WebGestalt KEGG pathway enrichment. Not surprisingly, the pathways for “Oxidative phosphorylation” and “TCA cycle”, important processes of cellular respiration, are among the top pathways enriched (**Table 4**). Given the importance of the pathways enriched for the *predominantly-on* class of genes, we make the general conclusion that the *predominantly-on* class is enriched for genes that are generally essential to cell survival and maintenance. Given their general importance and their tendency to be consistently expressed across a variety of tissue types, genes that fall into this GEP class are often referred to as “housekeeping genes”.

**Table 4:** Significantly enriched KEGG pathways for predominantly-on genes (*p*< = 0.01).

KEGG pathway	O: Genes observed	^1^E: Genes expected	^2^R: Ratio of enrichment	^3^p-Values< = 0.01
Oxidative phosphorylation	78	29.82	2.62	5.71e-24
Proteasome	25	7.68	3.25	1.52e-13
Citrate cycle (TCA cycle)	21	7.99	2.63	1.98e-7
Valine, leucine, and isoleucine degradation	29	12.91	2.25	3.55e-7
Reductive carboxylate cycle (CO2 fixation)	10	3.38	2.96	5.94e-5
N-Glycan biosynthesis	24	11.99	2.00	6.80e-5
Aminoacyl-tRNA biosynthesis	18	8.30	2.17	1.28e-4
Insulin signaling pathway	57	37.81	1.51	1.91e-4
SNARE interactions in vesicular transport	19	9.22	2.06	2.29e-4
Folate biosynthesis	20	10.14	1.97	3.68e-4
Tight junction	49	32.28	1.52	4.26e-4
Purine metabolism	57	39.04	1.46	5.28e-4
Chronic myeloid leukemia	35	21.52	1.63	5.73e-4
Ribosome	14	6.46	2.17	7.40e-4
Aminosugars metabolism	18	9.22	1.95	8.55e-4
Propanoate metabolism	16	7.99	2.00	1.13e-3
Long-term potentiation	29	17.52	1.66	1.16e-3
Pyrimidine metabolism	37	23.98	1.54	1.42e-3
Protein export	7	2.46	2.85	1.52e-3
Aminophosphonate metabolism	10	4.30	2.32	2.04e-3
Pentose phosphate pathway	15	7.68	1.95	2.31e-3
Pancreatic cancer	33	21.52	1.53	2.85e-3
Glycan structure - biosynthesis 1	42	29.20	1.44	3.78e-3
Fatty acid elongation in mitochondria	7	2.77	2.53	4.99e-3
Caprolactam degradation	8	3.38	2.37	5.06e-3
Basal transcription factors	16	8.91	1.79	5.32e-3
Cell cycle	44	31.35	1.40	5.35e-3
RNA polymrase	12	6.15	1.95	6.36e-3
Glioma	27	17.83	1.51	8.12e-3
Biosynthesis of steroids	9	4.30	2.09	9.84e-3

Column legend is the same as in **Table 2**.

### Pathway Enrichment for 2,387 Switch-Like Genes

**Figure 5:**
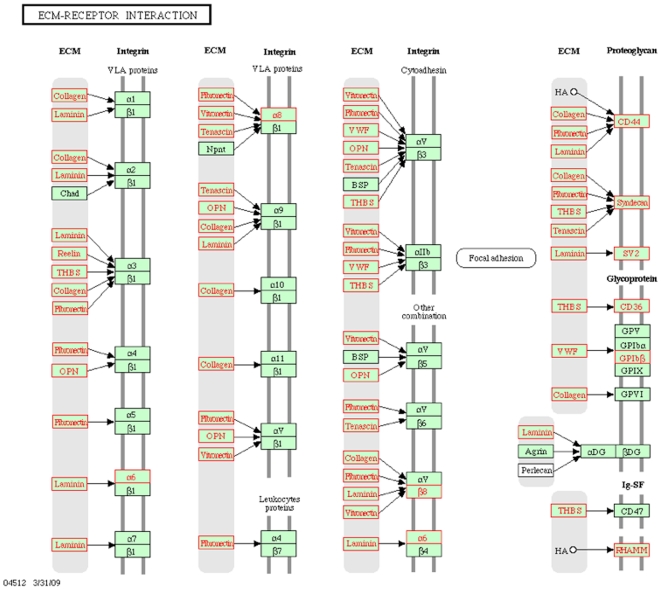
Switch-like genes highlighted in the KEGG “ECM-receptor interaction” diagram. Nodes representing switch-like genes are outlined in orange.

In cluster 4, the *switch-like* gene class, there are 2,387 known genes reported by WebGestalt for KEGG pathway enrichment analysis. Results for this class of genes were similar those obtained by Ertel and Tozeren on the study of bimodal *switch-like* genes via two-component mixture analysis [77]. “ECM-receptor interaction”, “Cell communication”, and “Focal adhesion” pathways identified by us (**Table 5**) were also identified among the top three significant pathways in that study (see **Table 3** of [77]). Thus, *switch-like* genes are enriched for functions in extracellular and intercellular signal transduction. For example, the α3/β1 integrin complex interacts with five extracellular proteins that are switch-like in the ECM-receptor interaction pathway (**Figure 5**).

**Table 5:** Significantly enriched KEGG pathways for switch-like genes (*p*< = 0.01).

KEGG pathway	O: Genes observed	^1^E: Genes expected	^2^R: Ratio of enrichment	^3^p-Values< = 0.01
Cell adhesion molecules (CAMs)*	46	17.01	2.70	3.76e-11
ECM-receptor interaction* $	37	12.17	3.04	5.43e-11
Cell Communication*	38	13.78	2.76	9.68e-10
Focal adhesion* $	55	25.22	2.18	6.42e-9
Leukocyte transendothelial migration*	38	14.66	2.59	7.58e-9
Cell cycle	38	14.96	2.54	1.43e-8
Complement and coagulation cascades	24	8.80	2.73	1.46e-6
Metabolism of xenobiotics by cytochrome p450	20	7.33	2.73	1.08e-5
PPAR signaling pathway* $	23	9.38	2.45	1.97e-5
Glutathione metabolism	14	5.13	2.73	2.26e-4
Type I diabetes mellitus*	14	5.57	2.51	6.12e-4
Hematopoietic cell lineage	21	11.00	1.91	2.05e-3
Glycolysis / Gluconeogenesis*	15	7.18	2.09	3.39e-3
B cell receptor signaling pathway	17	9.09	1.87	6.59e-3
Beta-Alanine metabolism	8	3.08	2.60	7.21e-3
Arginine and proline metabolism	13	6.45	2.02	8.56e-3
Urea cycle and metabolism of amino groups	8	3.23	2.48	9.90e-3

Column legend is the same as in **Table 2**.

* pathways which are also listed in **Table 2** of [78].

$ pathways which are also significantly enriched in both mouse and human data in the study by Ertel and Tozeren [77], [78].

Four of the seven significantly enriched KEGG pathways for switch-like genes found by Ertel and Tozeren (as seen in **Table 3** of [77]) are also among the 17 significantly enriched pathways found by us (**Table 5**). We used the same threshold of evidence against the null hypothesis (*p*-value< = 0.01) and identified more enriched KEGG pathways for switch-like genes as seen in **Table 5** than that study [77]. This is expected because we analyzed many more mouse arrays in our study (2145 vs. 388 mouse arrays). When both mouse and human bimodal genes found by Ertel and Tozeren were taken into account [77], [78], eight (marked with * in **Table 5**) of the 17 significantly enriched KEGG pathways found by us are also listed in Table 2 of [78], which lists significantly enriched pathways for human or mouse switch-like genes (or both).

Among the above-mentioned eight pathways (found by us), the following three pathways (marked with ^$^ in **Table 5**) are also significantly enriched in both mouse and human data by Ertel and Tozeren [77], [78]: 1) Focal adhesion, 2) PPAR signaling pathway, and 3) ECM-receptor interaction. Interestingly, five of these eight pathways (marked * in **Table 5**) are among the top five significantly enriched pathways listed in our study.

### Tissue-Specific Biomarker Genes

Tissue-specific biomarkers are important for diagnosing and monitoring disease. We leveraged our analysis of GEPs to identify tissue-specific biomarker genes. For example, 279 arrays hybridized with brain tissue were grouped separately from non-brain arrays. For each gene, the GEPs derived from 279 brain-specific arrays and those derived from 1866 of non-brain arrays were overlaid and compared. Potential brain biomarker candidate genes were expected to have a GEP in brain that was distinct from the GEP derived from non-brain samples.

For each gene, GEPs from the brain and non-brain groups were compared using the Kolmogorov-Smirnov distance (KS_d) [34], [35] and the Pearson correlation coefficient (Corr) [36], [37]. Genes with KS_d value more than 0.8 and Corr value less than 0.1 were identified as putative biomarkers for brain tissue. Biomarker candidate genes for lung, liver, embryo, heart, and small intestine are listed in **Table S3**.

#### *343 Biomarker Candidate Genes in Brain*

**Figure 6:**
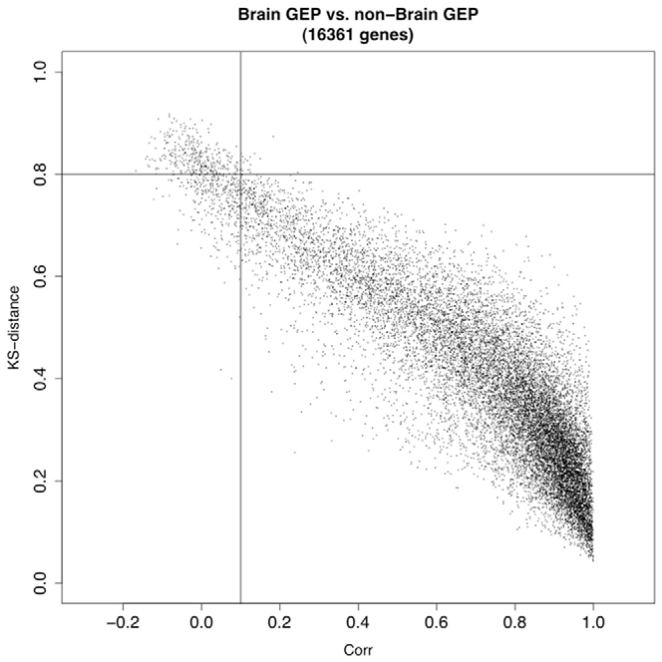
KS_d vs. Corr values for all GEPs. The horizontal line represents the cutoff KS_d>0.8; the vertical line represents the cutoff Corr <0.1. Potential biomarkers are in the box at upper left. Similar figures for lung, liver, embryo, heart, and small intestine are in **File S1**.

***Most of the GEPs among the 16361 analyzed array features from arrays hybridized with brain tissue are highly and positively correlated with the GEPs derived from the arrays hybridized with non-brain tissues*** (**Figure 6** **).** ***343 genes with KS_d>0.8 and Corr <0.1 were selected as candidate biomarkers. These cutoff thresholds were decided after many visual evaluations of the brain vs. non-brain GEPs; for candidate genes derived from various threshold choices, the GEP from the brain-tissue arrays was overlaid with the GEP from the non-brain arrays to visually evaluate how distinct the two GEPs are. The above thresholds were selected because most of resulting 343 biomarker candidate genes have GEPs which have GEP patterns that are clearly distinct in brain and non-brain, as determined by visual evaluation***.

The 343 biomarker candidate genes for brain were analyzed with WebGestalt for tissue expression pattern and gene enrichment analyses. WebGestalt's “Bar chart of the tissue expression pattern” (**Figure S5**) showed that brain tissue ranked first and included 317 out of the submitted 343 candidate genes (**Table S3**, Brain Biomarkers worksheet). According to the gene enrichment analysis for the KEGG pathway, the three most enriched pathways identified are related to brain function, i.e., the pathway groups “Neuroactive ligand-receptor interaction”, “Long-term depression”, and “Neurodegenerative disorders” (**Table 6**). Interestingly, some analyzed genes (data not shown) are known to be associated with “Alzheimer's disease”, “Parkinson's disease”, “Huntington's disease”, and “Prion disease,” all of which are related to brain tissue degeneration and damage. A directed acyclic graph of enriched GO terms for the 343 brain biomarker candidates is given in **Figure S6**. Of the remaining 26 genes (343 candidates minus 317 known to be expressed in brain according to WebGestalt), we are aware that at least 13 of these are likely false positives; these 13 are members of proteoglycan families, and the Affymetrix probesets used in this analysis do not distinguish between proteoglycans in the same family, owing to the identical 3′ ends of these family members. The remaining 13 candidates that were not identified by WebGestalt as expressed in brain are Locus 665113, 6430524H05Rik, A930034L06Rik, B930037P14Rik, BC068110, Bzrap1, Cacna2d3, Cntnap2, Dpysl4, Gprasp2, Lass1, Mast1, and Rprm.

**Table 6:** Three significantly enriched (*p*<0.05) KEGG pathways among 343 biomarker candidate genes for mouse brain.

KEGG pathway	Number of Genes	Entrez Gene IDs	Enrichment*
Long-term depression	7	14681 14687 14799 14800 269643 53623 72930	O = 7;E = 1.496;R = 4.6791;P = 7.26e-4
Neuroactive ligand-receptor interaction	12	108073 12801 14395 14400 14402 14406 14658 14799 14800 237213 53623 54393	O = 12;E = 5.2043;R = 2.3058;P = 6.30e-3
Neurodegenerative Disorders	3	11803 17762 22223	O = 3;E = 0.6532;R = 4.5928;P = 2.70e-2

* Abbreviations used in the “Enrichment” column: O: observed, E: expected, R: enrichment ratio, and P: hypergeometric *p*-value for enrichment.

Among the 20 biomarker candidate genes for human brain proposed by Laterza *et al*. in **Table 1** of [79], we found 10 genes in that table have the same or related matches among 343 biomarker candidate genes for mouse brain in our study, as seen in **Table 7**. The human brain biomarker genes proposed by Laterza *et al.* were homologues of mouse genes with high and specific expression in brain in their mouse array studies; 20 human homologues were confirmed afterward to be enriched in the human brain by the abundance of expressed sequence tags derived from a brain source in Unigene database [79]. In **Table 7**, the gene symbols in the left column are for the genes in **Table 1** of [79]; the gene symbols on the right are from our study where genes closely related to the matched gene were also included. This comparison confirms again the potential usefulness of these ten biomarkers for brain. Additionally, “GLRB, GLRA2” and “GABRG2, GABRA2, GABRB1, GABRB3” in **Table 7** are among the 12 biomarker candidates described below for the significantly enriched pathway “Neuroactive ligand-receptor interaction” in brain. We assess these genes to be good candidates for biomarkers (see below).

**Table 7:** Ten of 20 proposed human brain biomarker genes by [79] have the same or related matches among 343 biomarker candidate genes for mouse brain in our study.

Genes in Table 1 of [79]	Genes in 343 Biomarker Candidates of Mouse Brain
SNAP25	SNAP25, SNAP91.
SYT1	SYT4, SYT11.
FEZ1	FEZ1
GLRB	GLRB, GLRA2.
OLFM1	OLFM1
ZIC1	ZIC1
INA	INA
SLC32A1	SLC1A3, SLC6A1, SLC6A15, SLC22A17, SLC25A18, SLC35f1, SLC45A1.
SERPINI1	SERPINI1
GABRG2	GABRG2, GABRA2, GABRB1, GABRB3.

### ***12 Selected Brain Biomarker Candidate Genes in an Enriched KEGG Pathway***

Of the the 343 candidate genes, 330 of them (343 minus 13 likely false positives) have potential as good biomarkers for brain. Here, we narrow our attention to the significantly enriched pathway group “Neuroactive ligand-receptor interaction,” which has the largest number of brain-specific genes (12 genes) among the enriched pathways (**Table 8**).

**Figure 7:**
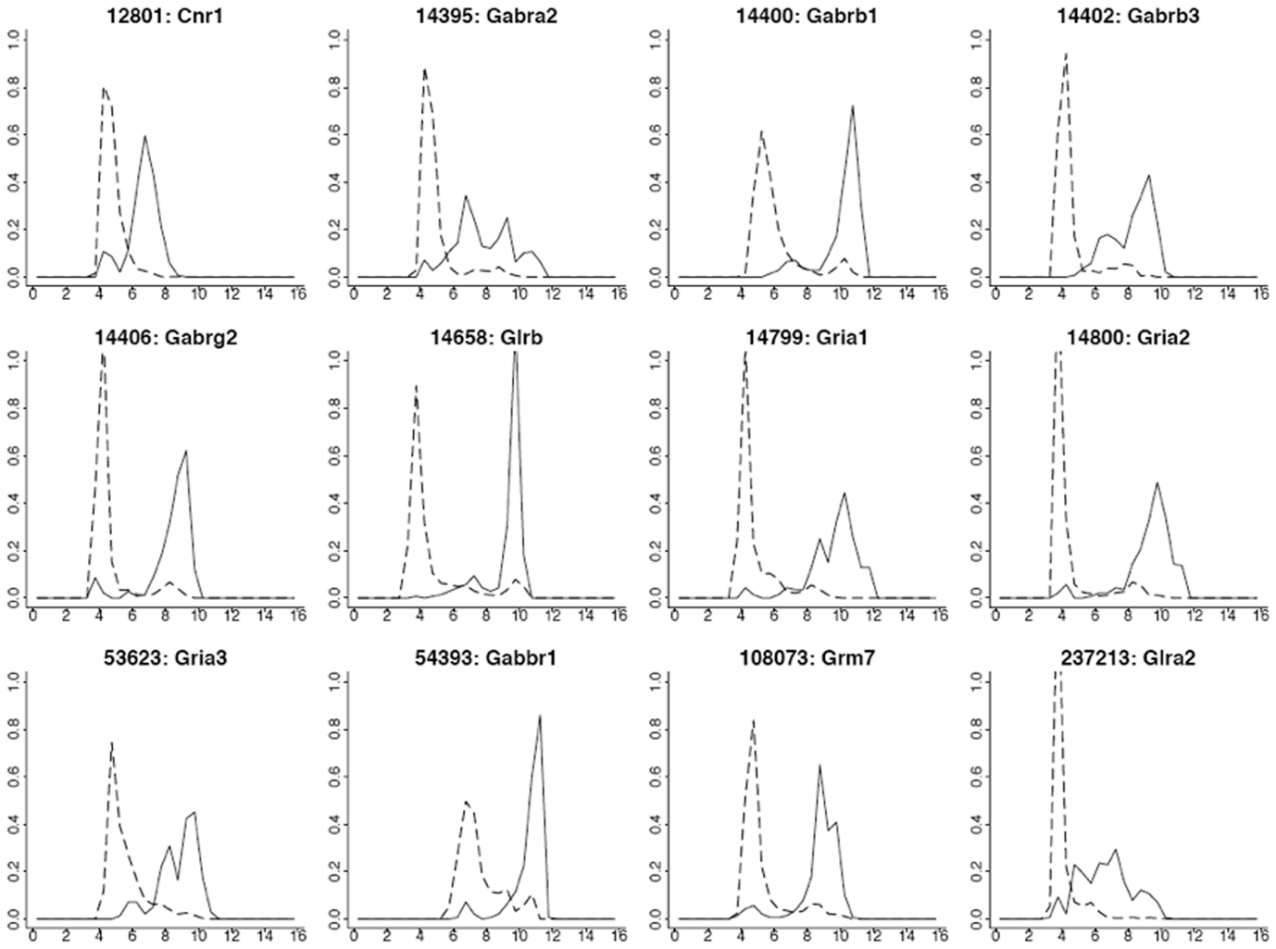
GEPs of the 12 candidate biomarkers from Table 8. Solid lines for brain specific GEPs, dashed lines for non-brain tissue GEPs. Log_2_ intensity values appear on the x-axis, probability density appears on the y-axis.

Most of the genes on the list in **Table 8** are switch-like genes belonging to cluster 4 (C4) as described previously. The GEPs of these 12 genes for brain-specific and non-brain tissues were overlaid in **Figure 7**. Most of these genes have weak expression intensity (around 2̂5 = 32) in non-brain tissues; also, most of the GEPs between brain-specific and non-brain tissues are different and clearly separated (i.e., switch-like) except the genes Cnr1, Gabra2, and Glra2 (**Figure 7**).

**Table 8:** Twelve strong candidate biomarker genes for mouse brain tissue.

Entrez ID	Unigene ID	Symbol	Gene Name
12801 (Cluster2)	Mm.7992	Cnr1	cannabinoid receptor 1 (brain)
14395 (Cluster4)	Mm.5304	Gabra2	gamma-aminobutyric acid (GABA-A) receptor, subunit alpha 2
14400 (Cluster4)	Mm.38567	Gabrb1	gamma-aminobutyric acid (GABA-A) receptor, subunit beta 1
14402 (Cluster4)	Mm.8004	Gabrb3	gamma-aminobutyric acid (GABA-A) receptor, subunit beta 3
14406 (Cluster4)	Mm.5309	Gabrg2	gamma-aminobutyric acid (GABA-A) receptor, subunit gamma 2
14658 (Cluster4)	Mm.275639	Glrb	glycine receptor, beta subunit
14799 (Cluster4)	Mm.4920	Gria1	glutamate receptor, ionotropic, AMPA1 (alpha 1)
14800 (Cluster4)	Mm.220224	Gria2	glutamate receptor, ionotropic, AMPA2 (alpha 2)
53623 (Cluster4)	Mm.327681	Gria3	glutamate receptor, ionotropic, AMPA3 (alpha 3)
54393 (Cluster4)	Mm.32191	Gabbr1	gamma-aminobutyric acid (GABA-B) receptor, 1
108073 (Cluster4)	Mm.240881	Grm7	glutamate receptor, metabotropic 7
237213 (Cluster1)	Mm.113877	Glra2	glycine receptor, alpha 2 subunit

The designations in the parentheses of “Entrez ID” column are for the gene category assignments as described in the text.

**Figure 8:**
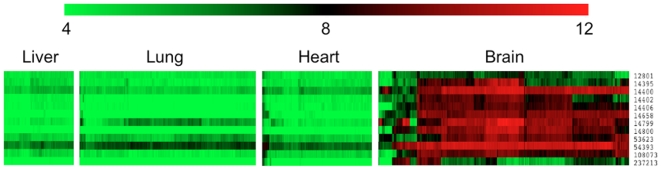
Heatmaps of log_2_ expression values of the twelve candidate genes from Table 8, in various tissues. Green is low, black is middle and red is high expression.

The gene expression heat maps for these 12 genes in the tissues of liver, lung, heart and brain were displayed in **Figure 8**. **Figures 7** **and** **8** show that the third to eleventh genes have very distinct expression profiles in brain as compared with other tissues. These genes thus have stronger potential to be good biomarkers for brain tissue.

There are three types of potentially good biomarkers identified here: the subunit genes for gamma-aminobutyric acid receptor (GABA_A_R and GABA_B_R), glycine receptor (GLR), and glutamate receptor. Subunit genes for both GABA_A_ and GABA_B_ receptors were identified in **Table 8**. GABA_A_ receptor is an ionotropic receptor and ligand-gated ion channel [80], [81]. GABA_B_ receptor is a metabotropic transmembrane receptor [82], [83]. Both receptors interact with gamma-aminobutyric acid (GABA), which is the chief inhibitory neurotransmitter in the mammalian central nervous system. Glycine receptor is the receptor for the amino acid neurotransmitter glycine; it is one of the most widely distributed inhibitory receptors in the central nervous system [84], [85]. Glutamate receptors bind glutamate, the most prominent neurotransmitter in the body, and are transmembrane receptors located on the membranes of neurons [86], [87]. Therefore, these three kinds of receptors have specialized functions in the central nervous system and the subunit genes for them can indeed be good biomarkers in the brain tissue. Subunit genes for GABA_A_R and GLR were also proposed by Laterza *et al.* (**Table 1** of [79]) as human brain biomarker genes. Although these biomarker gene products are the subunit proteins for assembly of the above-mentioned receptors, which are mostly transmembrane complexes, the subunit proteins of these receptor complexes can egress out of the brain to the peripheral blood when they are highly expressed in brain in a disease or cancer state. This is due to their size with a predicted protein sequence chain of *Mr* <70,000. The *Mr* 70,000 cutoff was selected because plasma albumin is known to enter the brain when blood-brain barrier was damaged after brain injury, suggesting this cutoff value for the egress of proteins out of the brain [79].

**Figure 9:**
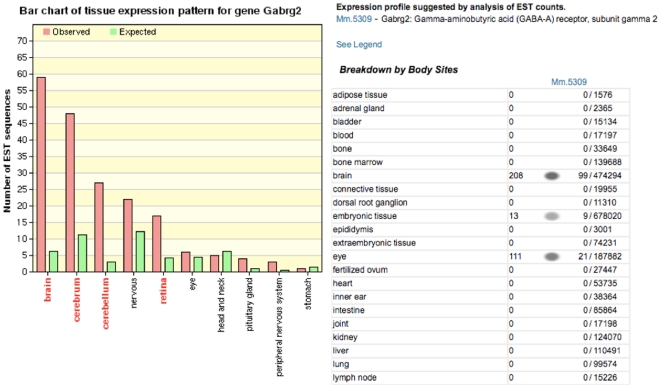
Tissue expression pattern and profiles for the gene Gabrg2. **Left**: (**a**) Tissue expression pattern for Gabrg2 (in **Table 8**) via WebGestalt. The height of the red bar represents the observed number of EST sequences for the selected gene in the tissue. The height of the green bar represents the expected number of EST sequences (Expected number of EST sequences for a specific gene in a specific tissue  =  Total number of EST sequences for the gene in all tissues x Total number of EST sequences for all genes in the tissue/Total number of EST sequences for all genes in all tissues). Tissue types in which the gene is significantly over-represented (*p*<0.01) are labeled red. Tissue types in which the gene is significantly under-represented (*p*<0.01) are labeled blue. **Right**: (**b**) Expression profile suggested by analysis of EST counts in UniGene database for the gene Gabrg2 (UniGene ID: Mm.5309); only portion (top part) of the profile is shown. The first column is for the pool name. The number on the left side of the spot column is in the unit of “Transcripts per million” (TPM) and the ratio on the right side of the spot column is the ratio of “EST of the query gene /Total EST in pool”. The spot intensity is based on TPM.

Another way to confirm the above 12 candidate genes as good biomarkers for brain tissue is to query the UniGene database on the tissue expression pattern of these genes based on the counts of Expressed Sequence Tags (ESTs) that have been sequenced to date. We examined Gabrg2 as an example. This gene is confirmed to be highly expressed in brain as shown in the tissue expression pattern created by WebGestalt with EST counts (**Figure 9a**) and the tissue expression levels seen with UniGene's EST ProfileViewer (**Figure 9b**). In the same way, most of the 12 genes in **Table 8** were confirmed to be highly expressed in brain, with the genes Gabra2, Gabrb1, Gabrg2, Gria1, Gria2, and Glra2 also highly expressed in spinal cord. The two exceptions in the EST profiles are Glrb (highly expressed in adrenal gland) and Gria3 (highly expressed in oviduct). In summary, 317 of the 343 candidate genes described above were found to be highly expressed in brain or nervous system by WebGestalt (**Figure S5**). This suggests that our methodology can be effectively used to automatically detect biomarker candidate genes among different tissues.

### Conclusion

It should be noted that assignment of cluster membership, as well as the comparison of expression profiles between a tissue of interest and it's complement in the dataset in this study are provisional on the limitations of the sample set used. Although a relatively large and heterogenous sample set was used, numerous tissues and cell types are un- or under-represented by this dataset.

We found that four distribution metrics (i.e., mean, standard deviation, kurtosis, and skewness) for GEPs can be used to sort genes into four gene classes: *predominantly-on*, *predominantly-off*, *graded*, and *switch-like*. Silhouette values for each gene were also used to refine the category (cluster) assignment for each gene. *Switch-like* genes discovered by us are similar to the switch-like genes identified by Ertel and Tozeren [77] with some differences likely attributable to differences in the number of array features and number of arrays used in each study.

Most of the identified candidate genes for the tissue-specific biomarkers were confirmed to have tissue-specific expression patterns by the tissue expression pattern constructed by WebGestalt and the tissue expression profile suggested by analysis of EST counts in the UniGene database. This suggests that our methodology can be used with minimum supervision for the identification of tissue-specific biomarker candidate genes, which may then be validated by mining experimental data or be confirmed by prospective experiments. The same strategy can be utilized in other datasets to identify putative biomarkers for specific disease states. Our new disease-state evaluation algorithm (based on the expression density distribution) will be useful for the discovery of disease-specific biomarker genes important for the diagnosis, treatment, or prognosis of related disease.

## Supporting Information

Figure S1 Frequency histograms of the distribution of four metrics for more than 16,000 GEPs. Upper panel: original distribution of metrics; lower panel: distribution of metrics after normalization and rescaling.
(0.15 MB PDF)

Figure S2 Distributions of silhouette values for the genes in each cluster.
(0.06 MB DOC)

Figure S3 Predominantly-off genes highlighted in KEGG “Linoleic acid metabolism” diagram. Nodes representing Predominantly-off genes are outlined in orange.
(0.03 MB DOC)

Figure S4 Graded genes highlighted in the KEGG “Taste Transduction” diagram. Nodes representing graded genes are outlined in orange.
(0.04 MB DOC)

Figure S5 Bar chart of the tissue expression pattern for 343 brain biomarker candidate genes.
(0.05 MB DOC)

Figure S6 Directed acyclic graph of Gene Ontology enrichment for 343 brain biomarker candidates.
(0.80 MB JPG)

Table S1 Affymetrix datasets used in this study (as in Jupiter and VanBuren, 2008) *** Note: Refer to .xls source file ***
(0.13 MB XLS)

Table S2 Distribution metrics and gene classes. *** Note: Refer to .xls source file *** This Excel Workbook contains a list of all CEL files used in the analysis (by GEO accession), the resulting raw and scaled distribution metrics for that data, and annotated lists of refined cluster (class) assignments.
(9.80 MB XLS)

Table S3 Biomarker tables. *** Note: Refer to .xls source file *** This Excel workbook contains metric data used to identify candidate biomarkers, and a list of the identified biomarkers for each tissue analyzed, including brain, lung, liver, embryo, heart, and small intestine.
(12.38 MB XLS)

File S1 This file contains supplementary text and figures, including supplementary methods, and figures that show the cutoffs for candidate biomarker identification.
(0.30 MB DOC)
