# Supplementary material for: Classification of Genes and Putative Biomarker Identification Using Distribution Metrics on Expression Profiles
Source: PLoS One. 2010 Feb 4;5(2):e9056. doi: 10.1371/journal.pone.0009056 (PMC2816221; doi:10.1371/journal.pone.0009056)
Supplement: Figure S1 — Frequency histograms of the distribution of four metrics for more than 16,000 GEPs. Upper panel: original distribution of metrics; lower panel: distribution of metrics after normalization and rescaling. (0.15 MB PDF) [file pone.0009056.s001.pdf]

Log_2 Transformation of Kurtosis and Normalized Scaling of Four Metrics

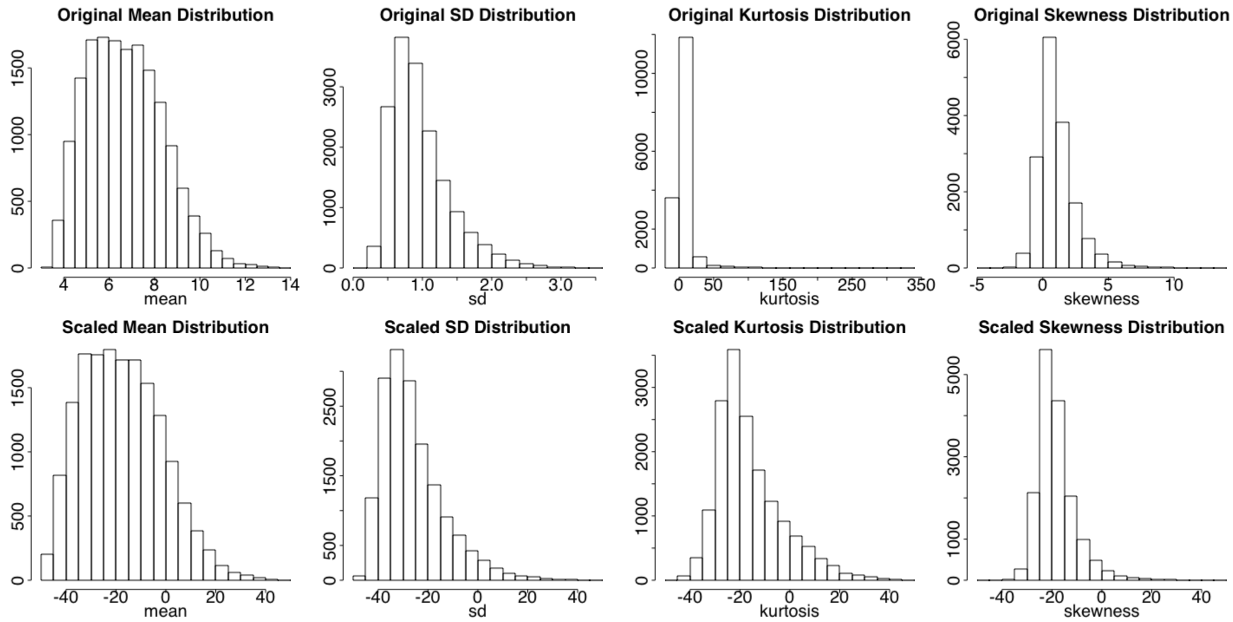

Figure S1. Frequency histograms of the distribution of four metrics for more than 16,000 GEPs. Upper panel: original distribution of metrics; lower panel: distribution of metrics after normalization and rescaling.
