# Supplementary material for: Classification of Genes and Putative Biomarker Identification Using Distribution Metrics on Expression Profiles
Source: PLoS One. 2010 Feb 4;5(2):e9056. doi: 10.1371/journal.pone.0009056 (PMC2816221; doi:10.1371/journal.pone.0009056)
Supplement: Figure S2 — Distributions of silhouette values for the genes in each cluster. (0.06 MB DOC) [file pone.0009056.s002.doc]

Refinement of Gene Categories by Silhouette Values of GEP Metrics


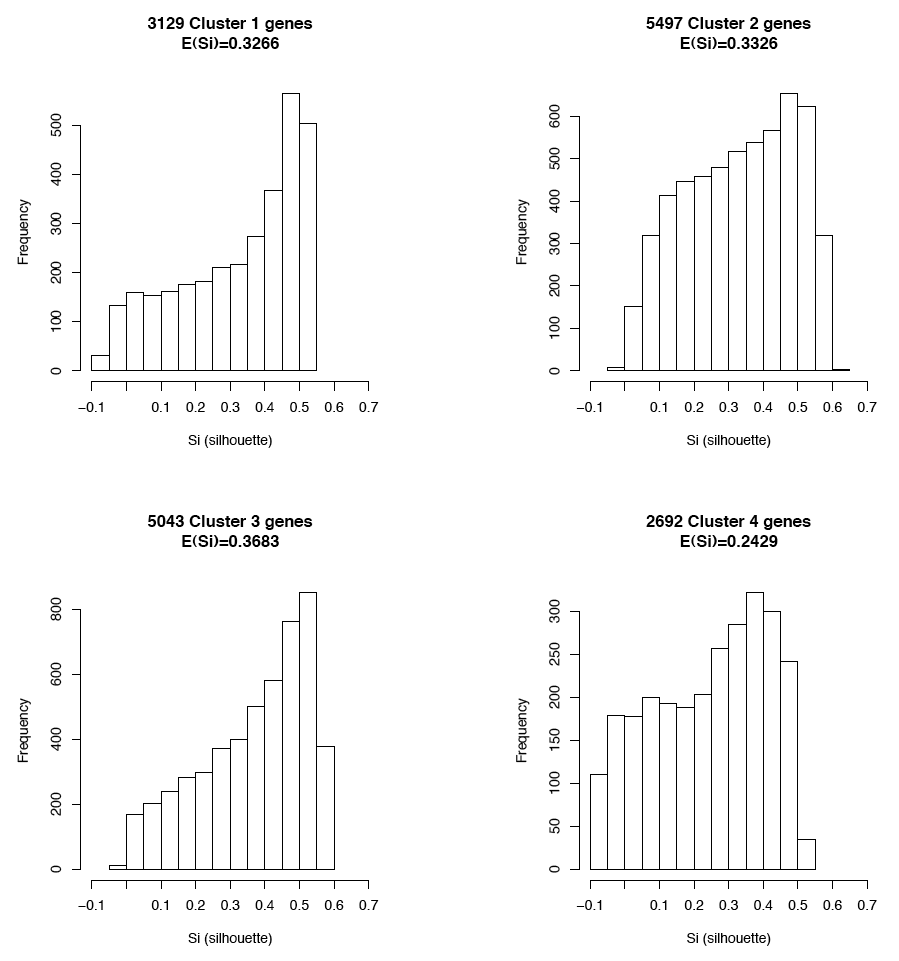


Figure S2. The distributions of the silhouette values for the genes in each cluster.
