# Supplementary material for: Classification of Genes and Putative Biomarker Identification Using Distribution Metrics on Expression Profiles
Source: PLoS One. 2010 Feb 4;5(2):e9056. doi: 10.1371/journal.pone.0009056 (PMC2816221; doi:10.1371/journal.pone.0009056)
Supplement: Figure S3 — Predominantly-off genes highlighted in KEGG “Linoleic acid metabolism” diagram. Nodes representing Predominantly-off genes are outlined in orange. (0.03 MB DOC) [file pone.0009056.s003.doc]

Pathway Enrichment for 2,944 Predominantly-Off Genes


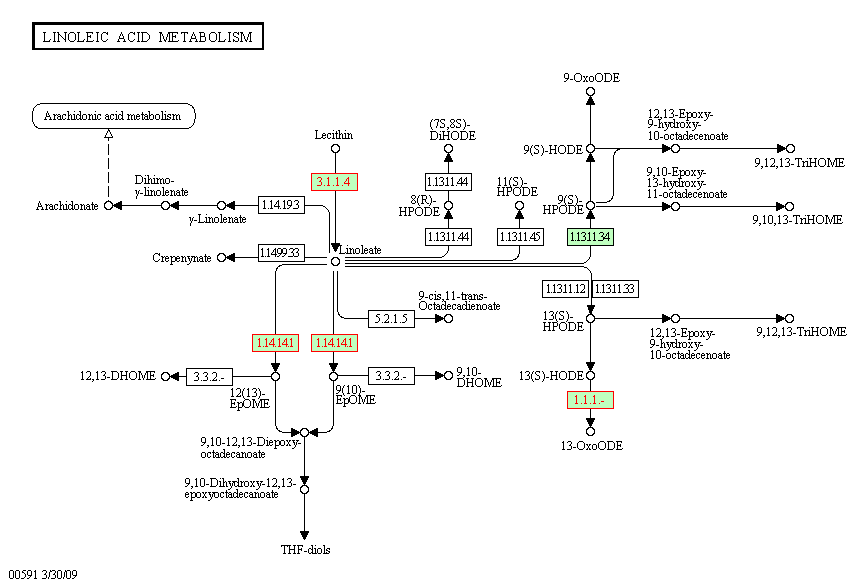


Figure S3. Predominantly-off genes highlighted in KEGG "Linoleic acid metabolism" diagram. Nodes representing Predominantly-off genes are outlined in orange.
