# Supplementary material for: Classification of Genes and Putative Biomarker Identification Using Distribution Metrics on Expression Profiles
Source: PLoS One. 2010 Feb 4;5(2):e9056. doi: 10.1371/journal.pone.0009056 (PMC2816221; doi:10.1371/journal.pone.0009056)
Supplement: Figure S4 — Graded genes highlighted in the KEGG “Taste Transduction” diagram. Nodes representing graded genes are outlined in orange. (0.04 MB DOC) [file pone.0009056.s004.doc]

Pathway Enrichment for 5,478 Graded Genes


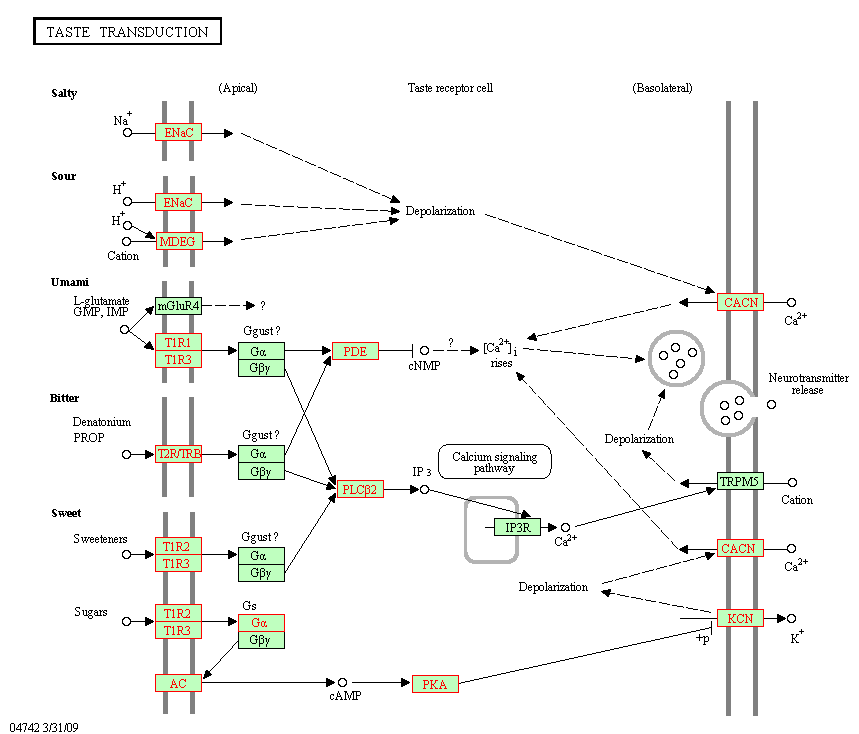


Figure S4. Graded genes highlighted in KEGG "Taste Transduction" diagram. Nodes representing graded genes are outlined in orange.
