# Supplementary material for: Classification of Genes and Putative Biomarker Identification Using Distribution Metrics on Expression Profiles
Source: PLoS One. 2010 Feb 4;5(2):e9056. doi: 10.1371/journal.pone.0009056 (PMC2816221; doi:10.1371/journal.pone.0009056)
Supplement: Figure S5 — Bar chart of the tissue expression pattern for 343 brain biomarker candidate genes. (0.05 MB DOC) [file pone.0009056.s005.doc]

343 Biomarker Candidate Genes in Brain


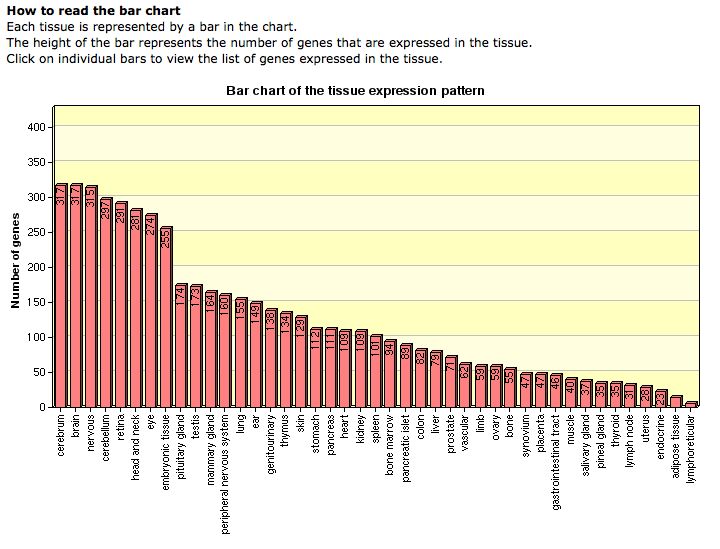


Figure S5. Bar chart of the tissue expression pattern for 343 brain biomarker candidate genes.
