# Supplementary figures and images for: Classification of Genes and Putative Biomarker Identification Using Distribution Metrics on Expression Profiles
Source: PLoS One. 2010 Feb 4;5(2):e9056. doi: 10.1371/journal.pone.0009056 (PMC2816221; doi:10.1371/journal.pone.0009056)

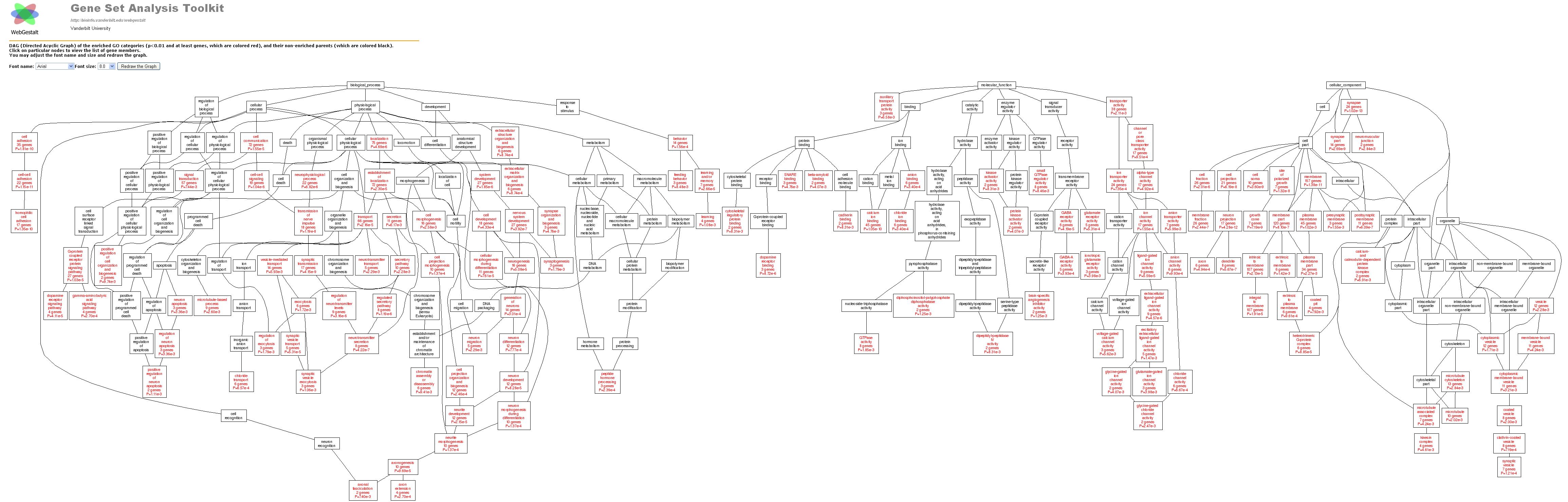

Supplement: Figure S6 — Directed acyclic graph of Gene Ontology enrichment for 343 brain biomarker candidates. (0.80 MB JPG) [file pone.0009056.s006.jpg]
