# Supplementary material for: Classification of Genes and Putative Biomarker Identification Using Distribution Metrics on Expression Profiles
Source: PLoS One. 2010 Feb 4;5(2):e9056. doi: 10.1371/journal.pone.0009056 (PMC2816221; doi:10.1371/journal.pone.0009056)
Supplement: File S1 — This file contains supplementary text and figures, including supplementary methods, and figures that show the cutoffs for candidate biomarker identification. (0.30 MB DOC) [file pone.0009056.s010.doc]

File S1

Methods

Silhouette Validation Method

The four metric values of mean, sd, kurtosis, and skewness for each gene's GEP were used to calculate the pairwise dissimilarity among the genes via Euclidean distance. The silhouette for a given gene is calculated as follows.

1). For each gene i, calculate the average dissimilarity a_i_ of gene i with other genes in its cluster.

2). For each gene i and each cluster l to which it does not belong, calculate the average dissimilarity b_il_ of gene i with members of cluster l.

3). Let b_i_ = min_l_ b_il_, then

the silhouette of gene i (S_i_) is defined by the following formula.

In this study, the silhouette measures how well matched a gene is to the other genes in its own cluster versus how well matched it would be if it were moved to the next closest cluster. It can be derived from the above formula that -1 ≤ S_i_ ≤ 1. If silhouette value is close to 1, it means the gene is "well-clustered". A silhouette near 0 indicates that a gene lies between two clusters, and a silhouette near -1 means that the gene is very similar to elements in the neighboring cluster and hence is probably in the wrong cluster.

Tissue-specific Biomarker Genes

Note: The cutoffs of the selection criteria for the biomarker candidate genes are arbitrary according to the study needs. For evaluation purposes, we used the cutoffs of KS-distance (KS_d) > 0.8 and Correlation Coefficient (Corr) < 0.1 to select the biomarker candidate genes for the tissues of Brain, Lung, Liver, Heart, and Small Intestine. For the Embryo tissue, we used the selection criteria of KS_d > 0.5 and Corr < 0.4 instead, due to the specialty of the Embryo tissue as seen in the KS_d vs. Corr plot.

462 Biomarker candidate genes for lung

Please see the Supplementary Excel file for the gene list.


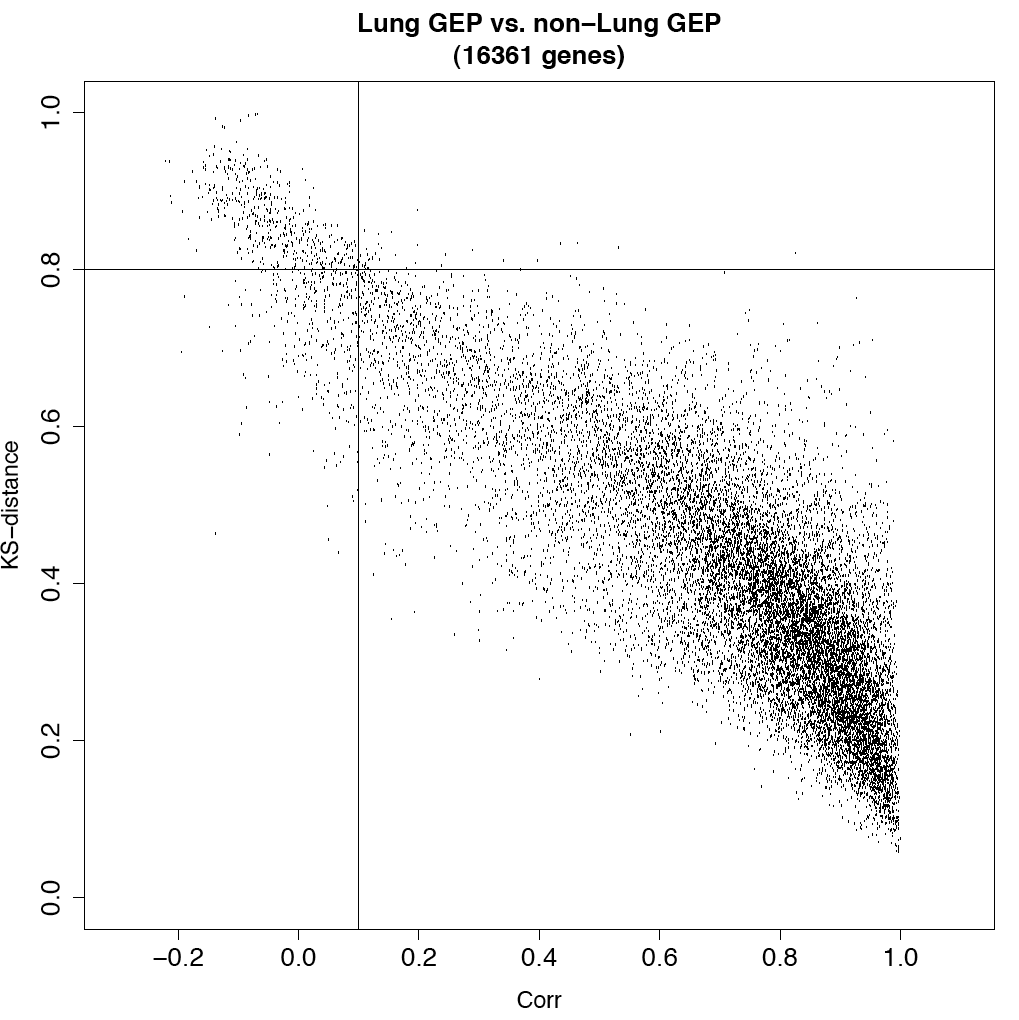


Lung vs. non-Lung GEPs for all genes: KS_d vs. Corr plot. The horizontal line represents the cutoff for KS_d > 0.8; the vertical line represents the cutoff for Corr < 0.1. Potential biomarkers are in the box at upper left.

258 Biomarker candidate genes for liver

Please see the Supplementary Excel file for the gene list.


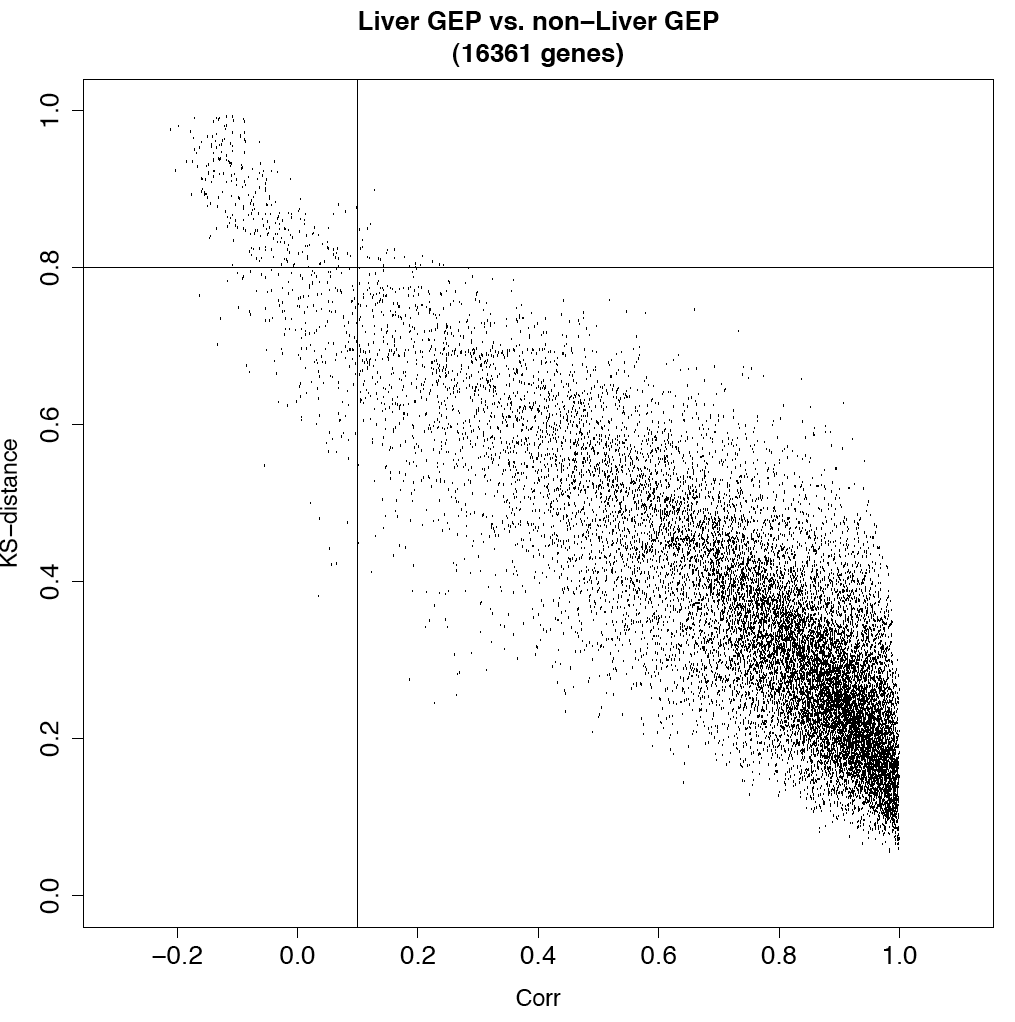


Liver vs. non-Liver GEPs for all genes: KS_d vs. Corr plot. The horizontal line represents the cutoff for KS_d > 0.8; the vertical line represents the cutoff for Corr < 0.1. Potential biomarkers are in the box at upper left.

191 Biomarker candidate genes for embryo

Please see the Supplementary Excel file for the gene list.


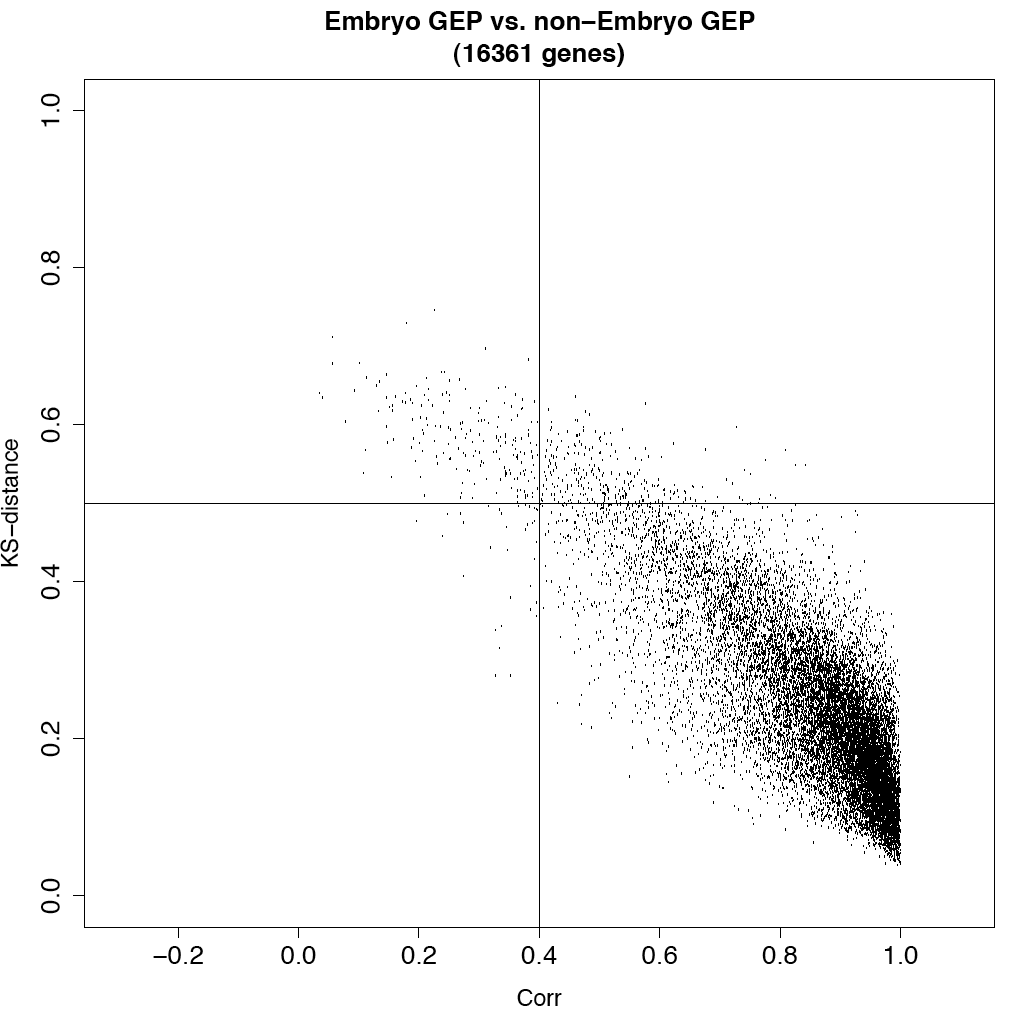


Embryo vs. non-Embryo GEPs for all genes: KS_d vs. Corr plot. The horizontal line represents the cutoff for KS_d > 0.5; the vertical line represents the cutoff for Corr < 0.4. Potential biomarkers are in the box at upper left. The cutoff criteria for Embryo biomarker candidates were loosened as seen from the above plot, due to the generic, universal, and multi-potent features of the genes in the embryonic cells when compared to the ones in other tissues.

201 Biomarker candidate genes for heart

Please see the Supplementary Excel file for the gene list.


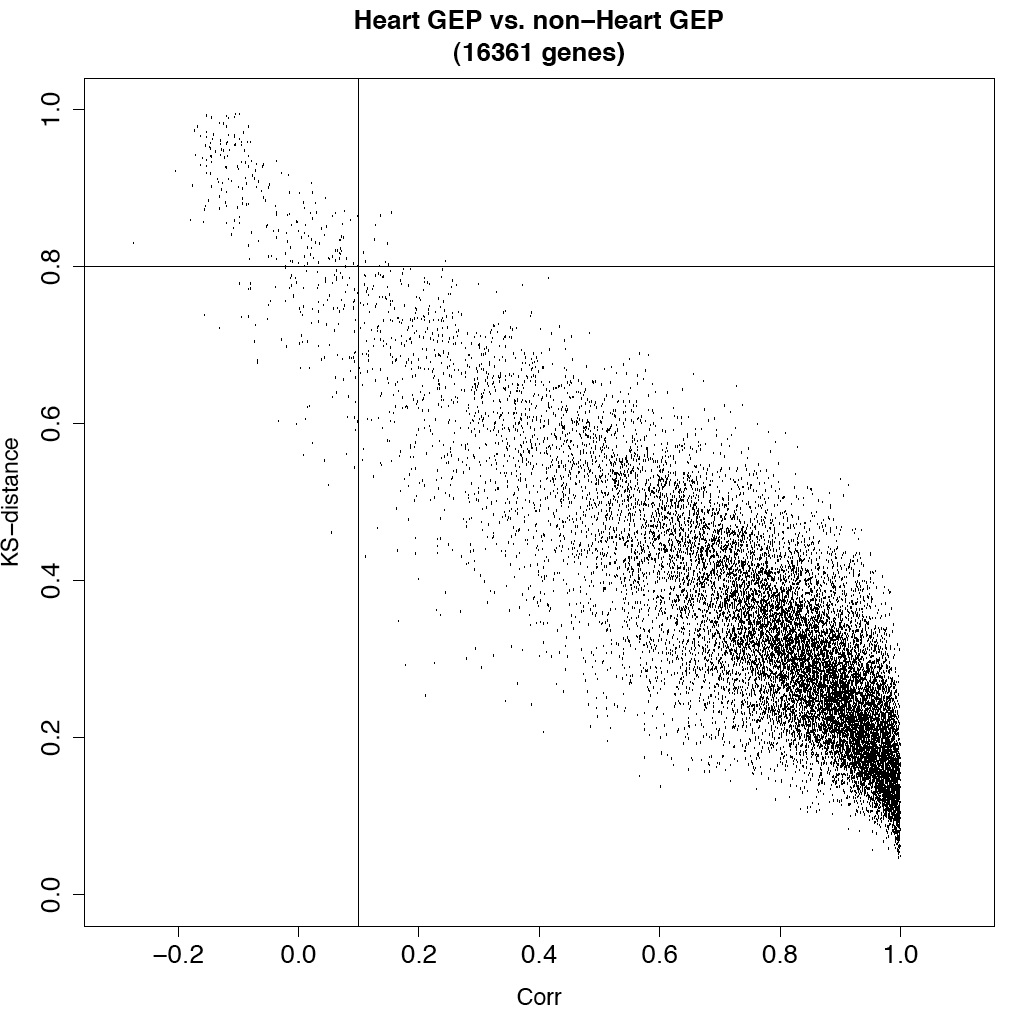


Heart vs. non-Heart GEPs for all genes: KS_d vs. Corr plot. The horizontal line represents the cutoff for KS_d > 0.8; the vertical line represents the cutoff for Corr < 0.1. Potential biomarkers are in the box at upper left.

146 Biomarker candidate genes for small intestine

Please see the Supplementary Excel file for the gene list.


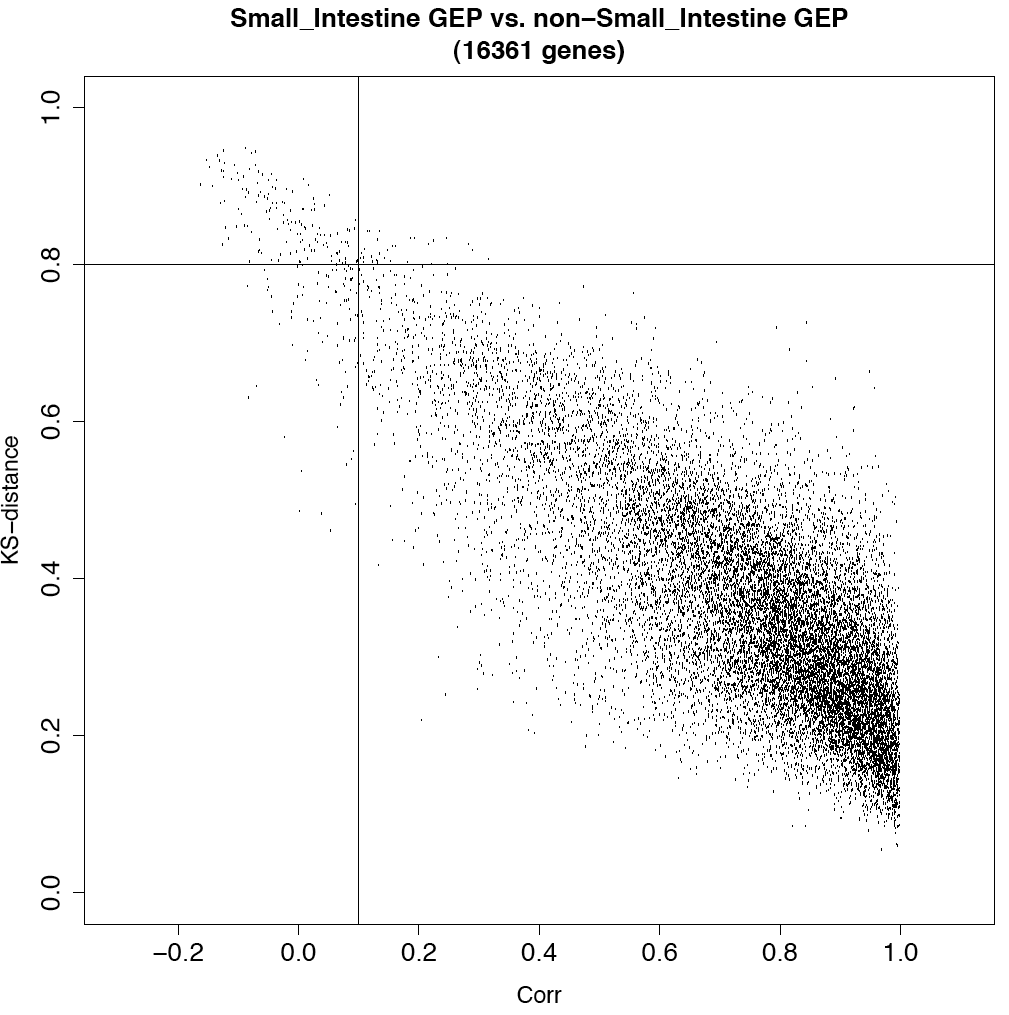


Small_Intestine vs. non-Small_Intestine GEPs for all genes: KS_d vs. Corr plot. The horizontal line represents the cutoff for KS_d > 0.8; the vertical line represents the cutoff for Corr < 0.1. Potential biomarkers are in the box at upper left.
